# Chitosan and Kappa-Carrageenan Vaginal Acyclovir Formulations for Prevention of Genital Herpes. *In Vitro* and *Ex Vivo* Evaluation

**DOI:** 10.3390/md13095976

**Published:** 2015-09-18

**Authors:** María-Pilar Sánchez-Sánchez, Araceli Martín-Illana, Roberto Ruiz-Caro, Paulina Bermejo, María-José Abad, Rubén Carro, Luis-Miguel Bedoya, Aitana Tamayo, Juan Rubio, Anxo Fernández-Ferreiro, Francisco Otero-Espinar, María-Dolores Veiga

**Affiliations:** 1 Departamento Farmacia y Tecnología Farmacéutica, Facultad de Farmacia, Universidad Complutense de Madrid, 28040-Madrid, Spain; E-Mails: mdelpilarss@gmail.com (M.-P.S.-S.); aracelimartin@ucm.es (A.M.-I.); rruizcar@ucm.es (R.R.-C.); 2 Departamento Farmacología, Facultad de Farmacia, Universidad Complutense de Madrid, 28040-Madrid, Spain; E-Mails: naber@ucm.es (P.B.); mjabad@farm.ucm.es (M.-J.A.); rubencarrohernan@gmail.com (R.C.); lmbedoya@ucm.es (L.-M.B.); 3 Instituto de Cerámica y Vidrio, Consejo Superior de Investigaciones Científicas, 28049-Madrid, Spain, E-Mails: aitanath@icv.csic.es (A.T.); jrubio@icv.csic.es (J.R.); 4 Departamento Farmacia y Tecnología Farmacéutica, Facultad de Farmacia, Universidad de Santiago de Compostela, Campus Vida s/n, 15782 Santiago de Compostela, Spain; E-Mails: anxordes@gmail.com (A.F.-F.); francisco.otero@usc.es (F.O.-E.)

**Keywords:** chitosan, kappa-carrageenan, vaginal mucoadhesive formulations, acyclovir controlled release, swelling behaviour, cytotoxicity, genital herpes, *ex vivo* bioadhesion

## Abstract

Vaginal formulations for the prevention of sexually transmitted infections are currently gaining importance in drug development. Polysaccharides, such as chitosan and carrageenan, which have good binding capacity with mucosal tissues, are now included in vaginal delivery systems. Marine polymer-based vaginal mucoadhesive solid formulations have been developed for the controlled release of acyclovir, which may prevent the sexual transmission of the herpes simplex virus. Drug release studies were carried out in two media: simulated vaginal fluid and simulated vaginal fluid/simulated seminal fluid mixture. The bioadhesive capacity and permanence time of the bioadhesion, the prepared compacts, and compacted granules were determined *ex vivo* using bovine vaginal mucosa as substrate. Swelling processes were quantified to confirm the release data. Biocompatibility was evaluated through *in vitro* cellular toxicity assays, and the results showed that acyclovir and the rest of the materials had no cytotoxicity at the maximum concentration tested. The mixture of hydroxyl-propyl-methyl-cellulose with chitosan- or kappa-carrageenan-originated mucoadhesive systems that presented a complete and sustained release of acyclovir for a period of 8–9 days in both media. Swelling data revealed the formation of optimal mixed chitosan/hydroxyl-propyl-methyl-cellulose gels which could be appropriated for the prevention of sexual transmission of HSV.

## 1. Introduction

Sexually transmitted infections (STIs) are a major global cause of acute illness, infertility, long-term disability, and death, with severe medical and psychological consequences for millions of men, women and infants.

WHO/Europe advocates and assists Member States in promoting and developing human-rights-based policies and practices for STI control and prevention. According to the World Health Organization, sexually transmitted diseases (STDs) and their complications are among the top five diseases in developing countries forcing patients to seek healthcare [1]. Neonatal diseases acquired by vertical transmission are serious complications associated with significant morbidity and mortality. STDs are also the second cause of disease-related death and loss of years of good health among young women of child-bearing age (excluding HIV).

Genital herpes is one of the most common sexually transmitted infections worldwide, with a global prevalence of 536 million people infected and an annual incidence of 23.6 million new cases [2,3]. This chronic disease is caused by the Herpes simplex virus (HSV) type 2, and presents a wide variability in its clinical manifestations, ranging from asymptomatic to mild or severe signs and symptoms with potential complications. There are consequently many non-diagnosed cases of genital HSV, as many people infected with HSV are unaware of their infection [4]. Over the last thirty years, epidemiologic and molecular studies have highlighted a strong and synergistic relationship between HSV-2 and the Human Immunodeficiency Virus-1 (HIV-1), which clearly points to their capacity for co-infection [5]. It should be noted that HSV-2 infection, even without recognized lesions, is an independent risk factor for HIV infection, such that the risk of HIV acquisition is three times higher in people with HSV-2. The resulting mucosal disruption caused by genital ulcers offers an effective entry route for HIV-1. This could be prevented by high concentrations of antiviral in the genital mucosa, thereby reducing the increased susceptibility to HIV-1 infection associated with HSV-2 [6,7,8,9]. While the probability of male-to-female transmission of STDs is alarmingly high, the same is not universally true for female-to-male transmission. Current methods of preventing STDs, such as abstinence, condoms, and monogamy are frequently ineffective and out of women’s control [10], making it advisable to design novel female-controlled barrier techniques, such as microbicides and female condoms [11]. Microbicides are currently emerging as a promising tool to protect women from STDs. A vaginal microbicide is any topical agent/formulation intended to prevent sexual pathogens, either by inactivating or killing cellular mechanisms, by forming a physical barrier between cells and pathogens, or by enhancing the natural protective mechanisms of the cervix and vagina. Unfortunately, many vaginal microbicide formulations may fail to elicit a protective response due to their lack of efficacy and inadequate formulation. Some of the most commonly used vaginal dosage forms include creams, gels, tablets, films, tampons, vaginal rings, and douches. Each of these formulations has specific advantages and limitations. Tablets can also be designed to perform a controlled-release of the microbicide over a prolonged period of time.

Several studies reveal that acyclovir (ACV) is a safe and effective drug for vaginal administration, and some clinical benefits have been observed in the treatment of primary or recurrent lesions from genital herpes [12,13]. Several studies on the prevention of genital herpes transmission have examined the inclusion of acyclovir as a microbicide drug in vaginal formulations such as gels [14], intravaginal rings [15,16], microporous matrices [17] or nanoparticles [18]. Vaginal bioadhesive tablets of acyclovir have been developed using different excipients such as methyl-cellulose, carboxy-methyl-cellulose, hydroxyl-propyl-cellulose, showing the dissolution results an inadequate behavior because of disintegration of tablets in the first 30 min. However, when hydroxyl-propyl-methyl-cellulose was incorporated to tablets, ACV release was prolonged during at least 8 h [19]. Gurumurthy *et al.* designed xanthan gum/Carbopol^®^ 934P-based acyclovir vaginal tablets obtaining sustained drug release data for 12 h in simulated vaginal fluid [20].

Mucoadhesive polymers have an excellent binding capacity with mucosal tissues over a considerable period of time. Several studies have been conducted on the incorporation of tragacanth, Carbopol^®^, Poloxamer 407^®^, pectin, sodium alginate, cellulose derivatives, and chitosan, among others, into vaginal formulations in order to increase the residence time of these formulations at the site of action [21,22,23].

Chitosan is a natural polysaccharide produced by the partial deacetylation of chitin, the structural element in the exoskeleton of crustaceans such as crabs and shrimps. The amino group in chitosan has a pKa value of approximately 6.5, which leads to protonation in an acidic solution with a density of charge dependent on the pH and the % deacetylation value. Chitosan has water soluble and bioadhesive properties with negatively charged surfaces, such as mucosal membranes, and can be used to transport a drug to an acidic environment where it can be degraded, thereby releasing the drug to the desired site. It is biocompatible and biodegradable and is widely used as a pharmaceutical excipient in a range of formulations such as powders, tablets, emulsions, and gels. The use of chitosan as a mucoadhesive polymer for vaginal delivery systems has been studied by several researchers [24,25,26].

Carrageenan is a member of the family of linear sulfated polysaccharides extracted from red edible seaweeds, which are widely used in the pharmaceutical industry for their gelling, thickening, and stabilizing properties. There are three main varieties of carrageenan, with differing degrees of sulfation. Kappa-carrageenan has one sulfate group per disaccharide, *iota*-carrageenan has two sulfates per disaccharide and *lambda* carrageenan has three. Liu *et al.* [27] developed an *in situ* kappa-carrageenan/poloxamer 407 vaginal gel with prolonged local residence. There is also evidence that carrageenan-based gel may offer some protection against HSV-2 transmission by binding with the receptors on the herpes virus, thus preventing the virus from binding to cells [28].

Cellulose derivatives have also been applied as drug delivery excipients in vaginal formulations. For instance, hydroxyl-ethyl-cellulose is a FDA-approved polymer found in a wide range of applications, because it is non-irritant and non-toxic to the vagina [29,30]. Methylcellulose has been used in the development of vaginal hydrogels due to its good biocompatibility and bioadhesion [31].

Other formulations, such as vaginal rings, gels or creams, vaginal tablets and compacts are easily manufactured, economical, stable under different environmental conditions, and easy to handle. If these solid formulations include the appropriate mucoadhesive polymer, or polymer mixture, an optimum formulation can be obtained for the *in situ* controlled release of the drug in the area where the transmission of vaginal herpes occurs.

With this background, the aim of this paper is to develop natural polymer-based vaginal mucoadhesive solid formulations for the controlled release of acyclovir, which may prevent the sexual transmission of HSV. Infection with HSV can increase the risk of infection with other pathogens, such as HIV.

## 2. Results and Discussion

In order to achieve optimal acyclovir controlled release mucoadhesive formulations, two types of solid systems were prepared containing 100 mg of acyclovir and natural and/or semisynthetic mucoadhesive polymers: compacts and compacted granules. Table 1 shows the composition (mg/unit) of these formulations.

**Table 1:** Composition (mg/unit) of prepared compacts and compacted granules.

Batch	Chitosan	*k*-Carrageenan	HPMC100	ACP	PVP	MgS	Acyclovir
CQ	225					3	100
CK		225				3	100
CH			225			3	100
CQH1	135		90			3	100
CQH2	90		135			3	100
CKH1		135	90			3	100
CKH2		90	135			3	100
CGQH1	135		90	45	27	3	100
CGQH2	90		135	45	27	3	100
CGKH1		135	90	45	27	3	100
CGKH2		90	135	45	27	3	100

### 2.1. Release Studies

**Figure 1:**
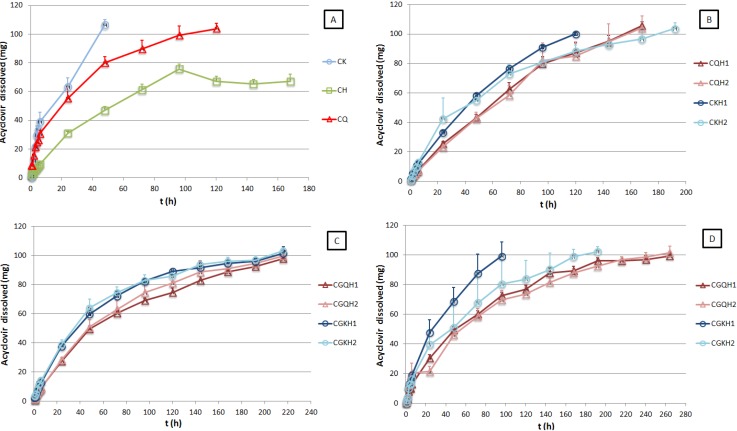
Acyclovir release profiles obtained from compacts and compacted granules in simulated vaginal fluid (**A**, **B**, and **C**) and simulated vaginal fluid/simulated seminal fluid mixture (**D**). All values represent the mean ± SD (*n* = 3).

Figure 1 shows the release profiles of acyclovir for all formulations assayed in simulated vaginal fluid (SVF) (graphs A, B, and C) and simulated vaginal fluid/simulated seminal fluid mixture (SVF/SSF) (graph D). The data from compacts prepared with polymer/acyclovir binary physical mixtures (CQ, CK, and CH) show that all the polymers control acyclovir release, but at different rates (graph A).

Kappa-carrageenan compacts (CK) produced drug release in 48 h, while samples of chitosan formulation (CQ) extend ACV release to 120 h. In both cases the swelling, disintegration, and dissolution of the formulation—the mechanisms controlling the release of A—were visually observed. Drug release was more prolonged when ACV was formulated with HPMC (CH), and the release was incomplete, as an asymptotic trend was observed in the release profile after 92 h of the test. At the conclusion of the release study it was observed that the CH compact core remained dry. The explanation is that in an aqueous medium HPMC forms a strong gel layer that prevents fluid from accessing the compact and hinders the dissolution of 100% of the dose of acyclovir. In the light of these findings, new formulations were designed combining a marine origin polymer such as chitosan or kappa-carrageenan with HPMC in order to exploit the benefits of both types of polymers. These include the swelling/dissolution—shown by the natural polymers studied—and the robustness of HPMC, which swells but will not dissolve in an aqueous medium. Figure 1 (graph B) shows the ACV release profiles obtained from the compacts formulated with the marine polymer/HPMC mixtures. As can be noted from the data, chitosan/HPMC compacts (CQH1 and CQH2) allow a complete and sustained release of ACV over a period of 168 h. Moreover, there are no appreciable differences between both profiles although they contain different chitosan/HPMC ratios. In contrast, kappa-carrageenan/HPMC compacts show total ACV release, but the time needed to achieve this depended on the kappa-carrageenan/HPMC ratio. The formulation containing the highest ratio of kappa-carrageenan (CKH1) released 100% of ACV in 120 h, whereas the CKH2 formulation required 192 h to release all of the drug. This may be the result of the ability of the kappa-carrageenan compacts to erode, as has been described by Bettini *et al.* [32] for the metoprolol/λ-carrageenan matrix. The chitosan-based compacts showed a strong interaction between chitosan and HPMC, and a more controlled release of ACV, regardless of the chitosan/HPMC ratio.

**Figure 2:**
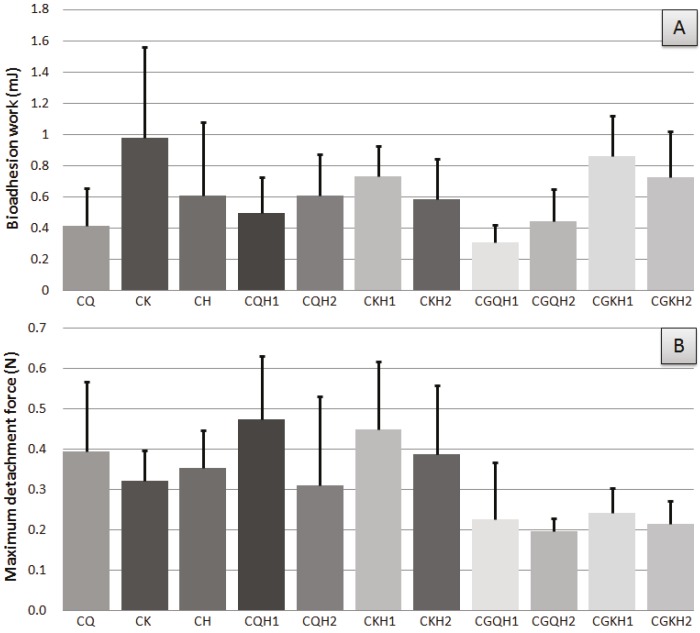
Vaginal bioadhesion work and maximum detachment force obtained for compacts and compacted granules (Mean Values + Standard Deviation, *n* = 6).

In order to prolong ACV release time, compacted granules were made by granulating physical ACV/polymer mixtures. Figure 1C shows the dissolution profiles of ACV for the compacted granule formulations. In all cases total drug release was obtained in a period of nine days (216 h). Although the systems containing the chitosan/HPMC mixture showed better controlled release of ACV, the granulation process can generally be assumed to lead to unitary systems (granules) where the presence of a binder (PVP K30) ensures the ACV+HPMC+marine polymer physical bond. The subsequent granule compaction produced robust formulations which enabled the sustained release of ACV. Since these formulations present superior ACV controlled-release characteristics, they were also tested in the simulated vaginal fluid/simulated seminal fluid mixture (SVF/SSF) to assess how the nature of the medium affected the ACV release process. The results of ACV released from CGQH1, CGQH2, CGKH1, and CGKH2 in the SVF/SSF mixture are shown in Figure 2D, and indicate that formulations with chitosan (CGQH1 and CGQH2) had a higher ACV controlled release. This is because pH-dependent chitosan dissolves better in an acidic medium (SVF) than in a neutral medium (SVF/SSF). Based on the results of the release studies, it can be concluded that chitosan-based systems may be the most suitable for use in preventing the sexual transmission of genital herpes. Although controlled ACV release was one of the aims of this research, it is also essential to verify whether the formulation has mucoadhesive properties. We, therefore, determined the bioadhesion of all the systems developed.

### 2.2. Characterization of Bioadhesion

The bioadhesive behavior of the compacts and compacted granules containing chitosan and kappa-carrageenan and HPMC are shown in Figure 2. All formulations show higher values for bioadhesion work and maximum detachment force in vaginal mucosa than for chitosan- and HPMC-based mucoadhesive acyclovir tablets in gastric mucosa [33], and for different mucoadhesive semisolid formulations (gel-type, such as Crinone, KYJelly, zidoval, and W/S mucoadhesive emulsions) [34].

The bioadhesive behavior of the compacts and compacted granules containing chitosan, kappa-carrageenan and/or HPMC are shown in Figure 2A. As expected, all the formulations had mucoadhesive ability.

The data on maximum detachment (separation) force for all the formulations evaluated are generally more homogeneous than the corresponding data for bioadhesion work (Figure 2B). This could indicate that once the samples have adhered to the mucosa, the detachment force is similar in all the formulations regardless of their composition, although a reverse behavior can be observed compared to the results for bioadhesion work. In general, greater force was required to separate each sample of chitosan-compacted formulations from vaginal mucosa: CQ > CK, CQH1 > CKH1 and CGQH1 > CGKH1. Moreover, the comparison between the detachment forces of compacted physical mixtures and compacted granule formulations (CQH1 *vs*. CGQH1, CQH2 *vs*. CGQH2, CKH1 *vs*. CGKH1, and CKH2 *vs*. CGKH2) clearly reveals a decrease in detachment forces for compacted granules with regard to the corresponding compacted physical mixtures; when PVP (binder agent) and ADCP (structural excipient) were included in the formulations, these excipients act as impurities that prevent the polymers from performing their mucoadhesive function.

### 2.3. Bioadhesion Residence Test

Once it was verified that all the prepared formulations had mucoadhesive properties, the next step was to determine how long they remained bonded to the vaginal mucosa. Table 2 shows the periods of time this adhesion lasted when formulations were immersed in SVF and SVF/SSF. CQ and CK samples remained adhered to the vaginal mucosa for less than 30 min in the case of simulated vaginal fluid, while CH samples remained for 120 h. The mucoadhesive ability of HPMC has been extensively verified, and appears to derive from its stronger hydrogen bonding with the mucin compared to other natural polymers [35,36]. Therefore the combination of HPMC with marine polymers (chitosan or kappa-carrageenan) may produce a longer attachment according to data obtained from both natural polymers. As expected, CQH1, CQH2, CKH1, and CKH2 remained adhered to mucosa for a longer period of time (between 72 and 108 h) than CQ and CK samples. The longer bonding time of CKH1 and CKH2 samples compared to CQH1 and CQH2 is due to kappa-carrageenan’s ability to form strong gels in the presence of potassium ions, which are part of the SVF composition. In other words, formulations that contain this polymer remain adhered to vaginal mucosa for longer than systems with chitosan. Chitosan is a polycationic copolymer consisting of glucosamine and *N*-acetylglucosamine units that are primarily responsible for swelling and the ensuing dissolution in contact with acidic fluid, and also have OH and NH_2_ groups that are considered essential for mucoadhesion [37].

**Table 2:** Bioadhesion residence time of acyclovir compacts and compacted granule formulations in simulated vaginal fluid (SVF) and simulated vaginal fluid/simulated seminal fluid (SVF/SSF) mixture.

Sample	Bioadhesion Residence Time in SVF	Bioadhesion Residence Time in SVF/SSF
CQ	<30 min	---
CK	<30 min	---
CH	120 h	---
CQH1	72 h	---
CQH2	72 h	---
CKH1	96 h	---
CKH2	108 h	---
CGQH1	72 h	72 h
CGQH2	72 h	96 h
CGKH1	72 h	72 h
CGKH2	72 h	72 h

The incorporation of a structural agent—anhydrous calcium hydrogen phosphate (ACP)—and a binder—polyvinyl pyrrolidone (PVP)—into the polymer blend to prepare wet granules produces compacted granules that remained adhered to the vaginal mucosa for the same period of time (72 h), regardless of the formulation composition or the pH of the medium (SFV or SVF/SSF mixture). This indicates that formulations obtained by combining HPMC with a natural polymer are reliable enough to remain adhered to the vaginal site for the time required to achieve ACV release, and consequently prevent the sexual transmission of genital herpes. If formulations were entirely composed of one polymer (chitosan, kappa-carrageenan or HPMC) they would have inappropriate mucoadhesion values, which may be deficient in the case of chitosan or kappa-carrageenan and excessive for HPMC.

### 2.4. Swelling Behavior

**Figure 3:**
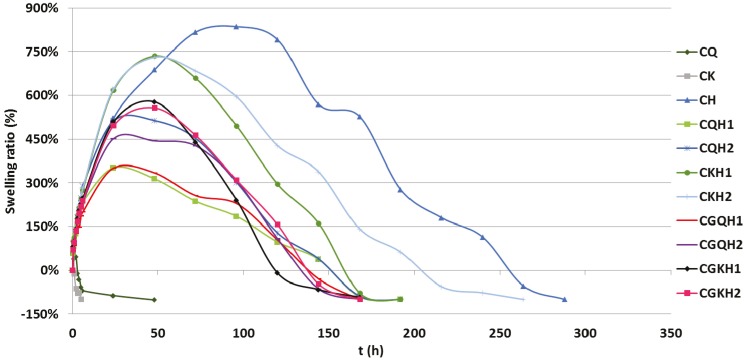
Swelling profiles obtained from compacts and compacted granules in simulated vaginal fluid.

Swelling processes were visually detected during release studies and mucoadhesion tests; however, it is very important to quantify this process, as the swelling curves can help explain the release results. Swelling ratio (SR) values obtained from all formulations determined in SVF are shown in Figure 3. Each positive SR value indicates that at a given time the swollen matrix weight was higher than that of the dry system weight (*t* = 0). Conversely, each negative SR value shows that the weight of the swollen system was lower than the weight of the dry system (*t* = 0). The swelling data for CQ and CK formulations reveal that both polymers are erodible in acidic media, while CH has the highest swelling profile. When HPMC is combined with chitosan or carrageenan, the formulations showed intermediate swelling, which would be more acceptable in terms of patient comfort. An analysis of all the formulations made with polymer mixtures showed that their swelling behavior was conditioned by two factors: the nature of the natural polymer (chitosan or kappa-carrageenan), and the type of formulation (compact or compacted granules). Compact formulations with chitosan—CQH1 and CQH2—displayed lower swelling values than the kappa-carrageenan-based samples (CKH1 and CKH2). The explanation is that the presence of chitosan prevents HPMC’s own swelling pattern, as it produces a mixed HPMC/chitosan gel. CKH1 and CKH2 had very similar swelling profiles for the first 48 h of the test, with a subsequent decrease in weight in inverse proportion to their HPMC content (CKH1 weight loss is greater than CKH2 weight loss), as kappa-carrageenan is unable to interact with HPMC. CKH1 and CKH2 swelling curves therefore have a similar shape to the CH swelling curve. The areas under the swelling curve are related to the amount of HPMC in each formulation.

The swelling behavior of all compacted granule formulations is very similar, as they all showed swelling peaks between 350% and 510%, although the chitosan-based formulations CGQH1 and CGQH2 had a lower maximum swelling (350% and 452%, respectively). ACP plays a key role in the swelling process in these formulations. In formulations with kappa-carrageenan (CGKH1 and CGKH2), ACP creates a structure that prevents the free swelling of HPMC. In formulations with chitosan, the ACP structure prevents the formation of the HPMC-chitosan mixed gel. In other words, ACP acts by modulating the swelling of the polymers, resulting in systems with a similar swelling ratio which are very different from those obtained from formulations without this carrier.

### 2.5. Cell Toxicity

The biocompatibility of the different formulations was evaluated through *in vitro* cellular toxicity assays. The toxicity of the components was studied by incubation at room temperature for 48 h at different concentrations before the assay to ensure that any potential toxic component of the materials would be present in the dilution. The cell culture was then treated with a suspension of the different dilutions. All the components were tested at a maximum concentration of 1000 μg/mL, except for chitosan, which was tested at 250 μg/mL due to solubility problems.

Experiments were performed on a lymphoblastic cell line (MT-2) to evaluate toxicity on the immune cells present in vaginal or uterine mucosae and in the uterus epithelial cell line (HEC-1A) to assess potential damage to mucosae integrity. CC_50_ were calculated for the drug or empty material when possible.

**Figure 4:**
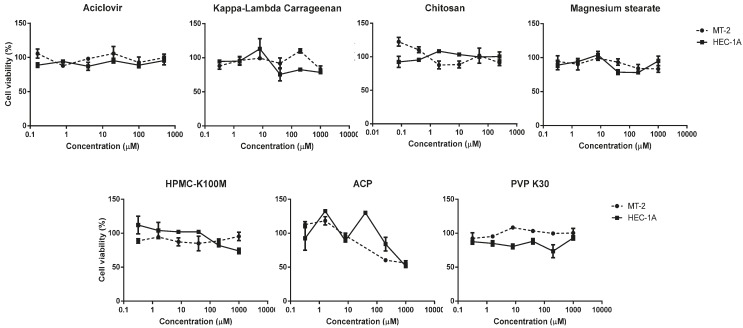
Graphic representation of the cytotoxic evaluation of acyclovir, chitosan, kappa-carrageenan, magnesium stearate, HPMC-K100M, ACP, and PVP K30. Cytotoxicity was measured in MT-2 cells and HEC-1A cells for 48 h. Cell viability is expressed as percentage of living cells compared to a non-treated control (100%).

As shown in Table 3 and Figure 4, no toxicity was detected at the concentrations tested except in the case of ACP, which showed mild toxicity in both cell lines, with a CC_50_ value of around 1000 μg/mL. Acyclovir and all other materials showed no cytotoxicity at the maximum concentration tested, so CC_50_ values were not calculated.

**Table 3:** CC_50_ values of acyclovir, chitosan, kappa-carrageenan, magnesium stearate, HPMC-K100M, ACP and PVP K30 in the cytotoxic assay in both MT-2 and HEC-1A cell lines. CC_50_: cytotoxic concentration 50%.

Drug	Cell Line	CC_50_
Acyclovir	MT-2	>500 μM
	HEC-1A	>500 μM
Chitosan	MT-2	>250 μg/mL
	HEC-1A	>250 μg/mL
Kappa-carrageenan	MT-2	>1000 μg/mL
	HEC-1A	>1000 μg/mL
Magnesium stearate	MT-2	>1000 μg/mL
	HEC-1A	>1000 μg/mL
HPMC-K100M	MT-2	>1000 μg/mL
	HEC-1A	>1000 μg/mL
ACP	MT-2	≈1000 µg/mL
	HEC-1A	≈1000 μg/mL
PVP	MT-2	>1000 μg/mL
	HEC-1A	>1000 μg/mL

## 3. Experimental Section

### 3.1. Materials

Acyclovir was obtained from Lab. Reig Jofré (Toledo, Spain). Chitosan with a deacetylation degree of 97% (CH, Lot: 8826900003) was supplied by Nessler (Madrid, Spain). Kappa-carrageenan (*k*; batch 088K0178) was provided by Sigma-Aldrich (St. Louis, MO, USA). Hydroxyl-propyl-methyl-cellulose Methocel^®^ K 100 M (HPMC; lot: DT352711) was a gift from Colorcon Ltd (Kent, UK). Anhydrous calcium hydrogen phosphate (ACP; batch: K93487944416) was provided by Merck (Darmstadt, Germany). Magnesium stearate PRS-CODEX (MGSt; batch: 85269 ALP) was purchased from Panreac (Barcelona, Spain). Kollidon^®^ 30 (PVP K30; batch: 98-0820) was supplied by BASF (Ludwingshafen, Germany). All other reagents in this study were of analytical grade and used without further purification. Demineralized water was used in all cases.

### 3.2. Preparation of Compacts and Compacted Granules

Two types of solid formulations, compacts and compacted granules, were prepared containing natural and/or semisynthetic mucoadhesive polymers, and 100 mg of acyclovir. This selected drug amount is based in a common application of a marketed vaginal formulation (acyclovir topical cream 5% *w*/*w*). The compacts (CQ, CK, CH, CQH1, CQH2, CKH1, and CKH2) were prepared from physical mixtures of the components, while the compacted granules (CGQH1, CGQH2, CGKH1, and CGKH2) were made from the corresponding granules in a pre-compacted stage. Granules were produced by adding a PVP K30 ethanol solution (binder agent) to a physical mixture of ACV, chitosan or kappa-carrageenan, HPMC, and ACP. ACP acts as structural agent. The wet mass was then passed through a 0.5 mm mesh and dried at 40 °C for 12 h. Magnesium stearate was added to the granules before compaction. Both types of systems were compacted according to the IR spectroscopy technique. A stainless steel disk was placed in a die assembly and the sample (physical mixture or granules) was added. A stainless steel disk was inserted in the cylinder bore on top of the sample. A piston was then placed inside the cavity, and the sample was pressed with a force of 5 t for 4 min. Finally, piston and disks were dismantled from the die and the acyclovir compact was removed and stored in a desiccator until use.

### 3.3. Release Study

All samples were placed in 100 mL DURAN laboratory borosilicate glass bottles containing 80 mL of release medium and stored in a shaking water bath (37 °C, 15 opm). Aliquots of 5 mL were withdrawn daily and filtered through Millipore^®^ filters (0.45 μm). The medium was replaced with an equal volume of release medium equilibrated to temperature. Acyclovir concentrations were quantified by UV spectroscopy at a wavelength of 251 nm in a Shimadzu^®^ UV-1700 spectrophotometer (Kyoto, Japan). The assay was made in triplicate in each case. In order to ensure that the formulations act as effective drug delivery carriers even in the presence of semen, the acyclovir release studies were carried out in two dissolution media: SVF (pH 4.2) and a SVF/SSF mixture (pH 7.6). In the first three hours of assay, the release medium was simulated vaginal fluid which was removed from each bottle and substituted by the SVF/SSF 1:4 *v*/*v* mixture until the end of the assay. Both media were prepared according to the methodology described in the bibliography [38,39].

### 3.4. Characterization of Bioadhesion

The bioadhesive capacity of the compacts and compacted granules was determined by measuring the maximum detachment force and the bioadhesion work using a TA-XT Plus texture analyzer (TA Instruments, Newcastle, UK), and bovine vaginal mucosa as substrate. Samples of bovine vaginal mucosa were obtained from a local slaughterhouse (Compostelana de Carnes S.L., Santiago de Compostela, Spain) immediately after slaughter. Each formulation was fixed to the upper support and the vaginal mucosa samples to the lower support using a cyanoacrylate adhesive. The mucosa was wetted with 0.5 mL of warm simulated vaginal fluid, and the system was equilibrated and maintained at 37 °C for 1 min. Finally a compression/extension stage was applied with an initial contact force of 0.5 N for 60 s to ensure close contact between the substrate and the sample, followed by an extension phase at a rate of 1 mm/s until the total separation of the components. The results were recorded as force *versus* elongation. Bioadhesion work was calculated as the area under the force-elongation curve. All the formulations were evaluated six-fold.

### 3.5. Bioadhesion Residence Test

A complementary *ex vivo* mucoadhesion test was conducted based on the rotating cylinder technique [26,40] to evaluate the permanence of the bioadhesion of the mucoadhesive formulations to the vaginal mucosa. A sample of freshly excised vaginal bovine mucosa obtained from a local slaughterhouse was fixed with acrylic adhesive to a stainless steel prism with a width of 2 cm anda height of 3 cm. In order to provide a homogeneous force of adhesion between the vaginal mucosa and the solid formulation, each sample was placed over the vaginal mucosa and pressed with a weight of 500 g for 30 s. The prism was then placed in a dissolution USP apparatus 1 (the basket was substituted by the prism) containing 500 mL of SVF at 37 ± 0.1 °C. The prism was fully immersed and agitated at 30 rpm. The time elapsed until the complete detachment, dissolution and/or erosion of the dosage forms was visually assessed. To determine whether the presence of semen influences the time the formulation remains attached to the vaginal mucosa, the bioadhesion remanence test was conducted under the same conditions but changing the nature of the medium. The samples were maintained for three hours in SVF and the medium was then exchanged for the SVF/SSF 1:4 *v*/*v* mixture [26]. Two replicates of each bioadhesion residence test were carried out in all cases.

### 3.6. Swelling Characterization

The influence of composition on the behavior of compacts and compacted granules was studied by determining the degree of swelling in simulated vaginal fluid according to a method described by Ruiz-Caro and Veiga [41]. Before the samples were exposed to the SFV they were glued to metallic discs with a diameter of 30 mm. The discs were then placed in beakers containing 100 mL of SFV. These beakers were placed in a thermostated water shaking bath (Selecta^®^ UNITRONIC320 OR, Barcelona, Spain) with an experimental temperature of 37 ± 0.1 °C and a shaking rate of 15 U/min. At specific time intervals, the samples were extracted from the test medium and blotted with filter paper to absorb the excess liquid on the sample surface before the weight evaluation. The swelling ratio (SR) of each sample was calculated according to the following equation: SR = ((Ts − Td)/Td) × 100, where Ts and Td were the weights of the swollen and dried samples respectively [42]. All swelling tests were performed in triplicate.

### 3.7. Cell and Cytotoxicity

Two human cell lines were used: a lymphoblastic cell line, MT-2 [43] and a uterus/endometrium epithelial cell line, HEC-1-A [44] (kindly provided by María Angeles Muñoz, Hospital Gregorio Marañón, Madrid). Both cells were grown and propagated in RPMI 1640 medium supplemented with 10% (*v*/*v*) foetal bovine serum, 2 mM L-glutamine and 50 μg/mL streptomycin at 37 °C with a humidified atmosphere of 5% CO_2_. The HEC-1-A cells were detached by removing the medium and rinsing the flask for 10 min with 1 to 2 mL of Trypsin 0.25%—EDTA 0.03% solution. The medium was replaced every third day after cell centrifugation at 1000 rpm for five minutes.

Cell toxicity was measured by the widely-used MTT (3-(4, 5-dimethylthylthiazol-2-yl)-2,5-diphenyltetrazolium bromide) method. Cells were incubated in 96-well plates at a density of 10 × 10^4^ cells per well in the case of MT-2, and 2 × 10^4^ in the case of HEC-1-A in complete medium. To assess the cytotoxic effects of acyclovir and the excipients used in the different formulations, cells were exposed to fresh medium containing various concentrations of compounds, or with the same concentration of vehicle to dissolved compound (DMSO) for 48 h as controls in triplicate. The particles were suspended in water following a standard method [45]. After 48 h of incubation, 20 μL of MTT solution (7.5 mg/mL) was added to the plate and incubated for another two hours for MT-2 cells and three hours for HEC-1-A cells. The supernatant was then carefully removed and 100 μL of dimethyl sulfoxide (DMSO) was added to each well. Absorbance at a wavelength of 550 nm was measured in a Labtech LT-4000 microplate spectrophotometer. Values of cytotoxic concentrations 50 (CC_50_) were calculated using GraphPad Prism Software (non-linear regression, log inhibitor *versus* response). The results of the MTT assay represent the average of at least three individual experiments.

## 4. Conclusions

From the results presented above it can be concluded that the combination of a polymer of marine origin—chitosan or kappa-carrageenan—with a semisynthetic polymer—hydroxyl-propyl-methyl-cellulose—in solid compacted formulations achieves the complete and sustained release of acyclovir over a period of between 220 and 260 h. These formulations can also adhere to the vaginal mucosa and remain adhered for 72 h. The swelling data of the formulations are influenced by the nature and quantity of the polymers they contain. Thus hydroxyl-propyl-methyl-cellulose has a higher degree of swelling than chitosan and kappa-carrageenan, which have lower swelling values and additionally are erodible in an acidic medium. The presence of anhydrous calcium hydrogen phosphate as a structural agent allows the modulation of the polymers’ swelling properties, reduces the swelling behavior of the hydroxyl-propyl-methyl-cellulose and prevents the formation of a mixed HPMC/chitosan gel. Of all the formulations developed, those containing chitosan are more indicated for preventing the sexual transmission of genital herpes, as added to their beneficial characteristics of mucoadhesion and sustained release of ACV, they showed a preventive effect of chitosan against cell damage.

## References

[1] Sexually Transmitted Infections. http://www.who.int/topics/sexually_transmitted_infection.

[2] Antoine T.E., Mishra Y.K., Trigilio J., Tiwari V., Adelung R., Shukla D. (2012). Prophylactic, therapeutic and neutralizing effects of zinc oxide tetrapod structures against herpes simplex virus type-2 infection. Antivir. Res..

[3] Cherpes T.L., Matthews D.B., Maryak A. (2012). Neonatal herpes simplex virus infection. Clin. Obstet. Gynecol..

[4] Garland S.M., Steben M. (2014). Genital herpes. Best Pract. Res. Clin. Obstet. Gynaecol..

[5] Tan D.H., Murphy K., Shah P., Walmsley S.L. (2013). Herpes simplex virus type 2 and HIV disease progression: A systematic review of observational studies. BMC Infect. Dis..

[6] Freeman E.E., Weiss H.A., Glynn J.R., Cross P.L., Whitworth J.A., Hayes R.J. (2006). Herpes simplex virus 2 infection increases HIV acquisition in men and women: Systematic review and meta-analysis of longitudinal studies. AIDS.

[7] Corey L., Wald A., Celum C.L., Quinn T.C. (2004). The effects of herpes simplex virus-2 on HIV-1 acquisition and transmission: A review of two overlapping epidemics. J. Acquir. Immune Defic. Syndr..

[8] Hen M., Heng S., Allen S. (1994). Co-infection and synergy of human immunodeficiency virus-1 and herpes simplex virus-1. Lancet.

[9] Moriuchi M., Moriuchi H., Williams R., Straus S.E. (2000). Herpes simplex virus infection induces replication of human immunodeficiency virus type 1. Virology.

[10] Podaralla S., Alt C., Shankar G.N. (2014). Formulation development and evaluation of innovative two-polymer (SR-2P) bioadhesive vaginal gel. AAPS PharmSciTech.

[11] Garg S., Tambwekar K.R., Vermani K., Kandarapu R., Garg A., Waller D.P., Zaneveld L.J.D. (2003). Development pharmaceutics of microbicide formulations. Part II: Formulation, evaluation, and challenges. AIDS Patient Care STDS.

[12] Corey L., Benedetti J.K., Critchlow C.W., Remington M.R., Winter C.A., Fahnlander A.L., Smith K., Salter D.L., Keeney R.E., Davis L.G. (1982). Double-blind controlled trial of topical acyclovir in genital herpes simplex virus infections. Am. J. Med..

[13] Corey L., Nahmias A.J., Guinan M.E., Benedetti J.K., Critchlow C.W., Holmes K.K. (1982). A trial of topical acyclovir in genital herpes simplex virus infections. N. Engl. J. Med..

[14] Shankar G.N., Alt C. (2014). Prophylactic treatment with a novel bioadhesive gel formulation containing acyclovir and tenofovir protects from HSV-2 infection. J. Antimicrob. Chemother..

[15] Moss J.A., Malone A.M., Smith T.J., Kennedy S., Kopin E., Nguyen C., Gilman J., Butkyavichene I., Vincent K.L., Motamedi M. (2012). Simultaneous delivery of tenofovir and acyclovir via an intravaginal ring. Antimicrob. Agents Chemother..

[16] Baum M.M., Butkyavichene I., Gilman J., Kennedy S., Kopin E., Malone A.M., Nguyen C., Smith T.J., Friend D.R., Clark M.R. (2012). An intravaginal ring for the simultaneous delivery of multiple drugs. J. Pharm. Sci..

[17] Asvadi N.H., Dang N.T.T., Davis-Poynter N., Coombes A.G.A. (2013). Evaluation of microporous polycaprolactone matrices for controlled delivery of antiviral microbicides to the female genital tract. J. Mater. Sci. Mater. Med..

[18] Ensign L.M., Tang B.C., Wang Y.Y., Tse T.A., Hoen T., Cone R., Hanes J. (2012). Mucus-penetrating nanoparticles for vaginal drug delivery protect against herpes simplex virus. Sci. Transl. Med..

[19] Genç L., Oğuzlar C., Güler E. (2000). Studies on vaginal bioadhesive tablets of acyclovir. Pharmazie.

[20] Gurumurthy V., Deveswaran R., Bharath S., Basavaraj B.V., Madhavan V. (2013). Design and optimization of bioadhesive vaginal tablets of acyclovir. Ind. J. Pharm. Educ. Res..

[21] Andersen T., Vanic Z., Flaten G.E., Mattsson S., Tho I., Skalko-Basnet N. (2013). Pectosomes and chitosomes as delivery systems for metronidazole: The one-pot preparation method. Pharmaceutics.

[22] Berginc K., Suljakovic S., Skalko-Basnet N., Kristl A. (2014). Mucoadhesive liposomes as new formulation for vaginal delivery of curcumin. Eur. J. Pharm. Biopharm..

[23] Li C., Han C., Zhu Y., Lu W., Li Q., Liu Y. (2014). *In vivo* evaluation of an in-situ hydrogel system for vaginal administration. Pharmazie.

[24] Gavini E., Sanna V., Juliano C., Bonferoni M.C., Giunchedi P. (2002). Mucoadhesive vaginal tablets as veterinary delivery system for the controlled release of an antimicrobial drug, acriflavine. AAPS PharmSciTech.

[25] Perioli L., Ambrogi V., Pagano C., Scuota S., Rossi C. (2009). FG90 chitosan as a new polymer for metronidazole mucoadhesive tablets for vaginal administration. Int. J. Pharm..

[26] Kast C.E., Valenta C., Leopold M., Bernkop-Schnürch A. (2002). Design and *in vitro* evaluation of a novel bioadhesive vaginal drug delivery system for clotrimazole. J. Control. Release.

[27] Liu Y., Zhu Y., Wei G., Lu W. (2009). Effect of carrageenan on poloxamer-based *in situ* gel for vaginal use: Improved *in vitro* and *in vivo* sustained-release properties. Eur. J. Pharm. Sci..

[28] Fernández-Romero J.A., Abraham C.J., Rodriguez A., Kizima L., Jean-Pierre N., Menon R., Begay O., Seidor S., Ford B.E., Gil P.I. (2012). Zinc acetate/carrageenan gels exhibit potent activity *in vivo* against high-dose herpes simplex virus 2 vaginal and rectal challenge. Antimicrob. Agents Chemother..

[29] Mahalingam A., Smith E., Fabian J., Damian FR., Peters J.J., Clark M.R., Friend D.R., Katz D.F., Kiser P.F. (2010). Design of a semisolid vaginal microbicide gel by relating composition to properties and performance. Pharm. Res..

[30] Yang S., Chen Y., Gu K., Dash A., Sayre C.L., Davies N.M., Ho E.A. (2013). Novel intravaginalnanomedicine for the targeted delivery of saquinavir to CD4+ immune cells. Int. J. Nanomed..

[31] Li N., Yu M., Deng L., Yang J., Dong A. (2012). Thermosensitive hydrogel of hydrophobically-modified methylcellulose for intravaginal drug delivery. J. Mater. Sci. Mater. Med..

[32] Bettini R., Bonferoni M.C., Colombo P., Zanelotti L., Caramella C. (2014). Drug release kinetics and front movement in matrix tablets containing diltiazem or metoprolol/λ-carrageenan complexes. Biomed. Res. Int..

[33] Ruiz-Caro R., Gago-Guillán M., Otero-Espinar F.J., Veiga M.D. (2012). Mucoadhesive tablets for controlled release of Acyclovir. Chem. Pharm. Bull..

[34] Campaña-Seoane M.J., Otero-Espinar F.J. Nuevas emulsiones mucoadhesivas para la liberación controlada de progesterona. Proceedings of the X Congreso de la Sociedad Española de Farmacia Industrial y Galénica.

[35] Mankala S.K., Korla A.C., Gade S. (2011). Development and evaluation of aceclofenac loaded mucoadhesive microcapsules. J. Adv. Pharm. Technol. Res..

[36] Tuğcu-Demiröz F., Acartürk F., Erdoğan D. (2013). Development of long-acting bioadhesive vaginal gels of oxybutynin: Formulation, *in vitro* and *in vivo* evaluations. Int. J. Pharm..

[37] Valenta C. (2005). The use of mucoadhesive polymers in vaginal drug delivery. Adv. Drug Deliv. Rev..

[38] Owen D.H., Katz D.F. (1999). A vaginal fluid simulant. Contraception.

[39] Owen D.H., Katz D.F. (2005). A review of the physical and chemical properties of human semen and the formulation of a semen simulant. J. Androl..

[40] Bernkop-Schnürch A., Steininger S. (2000). Synthesis and characterization of mucoadhesive thiolated polymers. Int. J. Pharm..

[41] Ruiz-Caro R., Veiga M.D. (2009). Characterization and dissolution study of chitosan freeze-dried systems for drug controlled release. Molecules.

[42] Haupt S., Zioni T., Gati I., Kleinstern J., Rubinstein A. (2006). Luminal delivery and dosing considerations of local celecoxib administration to colorectal cancer. Eur. J. Pharm. Sci..

[43] Harada S., Koyanagi Y., Yamamoto N. (1985). Infection of HTLV-III/LAV in HTLV-I-carrying cells MT-2 and MT-4 and application in a plaque assay. Science.

[44] Kuramoto H. (1972). Studies of the growth and cytogenetic properties of human endometrial adenocarcinoma in culture and its development into an established line. Acta Obstet. Gynaecol. Jpn..

[45] Krug H.F. (2011). Quality Handbook: Standard Procedures for Nanoparticle Testing.

